# Elevated mid-pregnancy plasma levels of angiotensin-converting enzyme 2 in women prior to the development of preeclampsia

**DOI:** 10.1038/s41598-022-08081-8

**Published:** 2022-03-08

**Authors:** Katja Junus, Inger Björk Ragnarsdóttir, Paliz Nordlöf Callbo, Lina Bergman, Susanne Lager, Anna-Karin Wikström

**Affiliations:** 1 Department of Women’s and Children’s Health, Uppsala University, Akademiska sjukhuset, 751 85 Uppsala, Sweden; 2 Department of Obstetrics and Gynecology, Institute of Clinical Sciences, Sahlgrenska Academy, University of Gothenburg, Gothenburg, Sweden; 3 Department of Obstetrics and Gynecology, Region Västra Götaland, Sahlgrenska University Hospital, Gothenburg, Sweden; 4 Department of Obstetrics and Gynecology, Stellenbosch University, Cape Town, South Africa

**Keywords:** Reproductive disorders, Biomarkers

## Abstract

Preeclampsia and cardiovascular disease (CVD) share multiple features and risk factors. Circulating angiotensin-converting enzyme 2 (ACE2) is increased in CVD and mediates SARS-CoV-2 entry into host cells, causing COVID-19 infection. The role of ACE2 in preeclampsia pathophysiology is unknown. We hypothesized that circulating ACE2 is increased in mid-pregnancy in women later developing preeclampsia. We included 296 women later developing preeclampsia (cases) and 333 women with a continuous healthy pregnancy (controls). Circulating ACE2 was measured with an immunoassay based on proximity extension assay technology, with levels being expressed as relative quantification on a log2 scale. Median (interquartile range) ACE2 levels were higher in cases than in controls; 3.84 (3.50–4.24) vs. 3.72 (3.45–4.04), *p* = 0.002. Adjusted logistic regression models showed a 60% increased risk for later development of preeclampsia with one unit elevation of ACE2 (adjusted odds ratio (aOR) 1.60, 95% confidence intervals (CI) 1.17–2.18). Preterm preeclampsia (diagnosis before 37 gestational weeks, *n* = 97) seemed to have a stronger ACE2 association than term preeclampsia, *n* = 199 (aORs, 95% Cis 2.14, 1.15–3.96 and 1.52, 1.04–2.23, respectively). Circulating ACE2 is increased at mid-pregnancy in women later developing preeclampsia, particularly preterm preeclampsia. Thus, our finding indicates a partly shared pathophysiological pathway between preeclampsia and CVD.

## Introduction

Preeclampsia is a heterogeneous syndrome characterized by new-onset hypertension and organ dysfunction after 20 gestational weeks. This syndrome can be devastating for both mother and child, being a leading cause of maternal and infant morbidity and mortality worldwide^1^. The pathophysiology of preeclampsia is complex, partially unknown, and may differ between phenotypes of the syndrome. Preeclampsia is characterized by endothelial dysfunction, oxidative stress, and systemic inflammation. In severe cases, women present with coagulopathy, severe hypertension, and end-organ complications. Preeclampsia predisposes for future cardiovascular disease (CVD), often presenting later in life, yet sometimes emerging soon after pregnancy^2^. The association with later CVD is stronger in preeclampsia with a preterm onset than preeclampsia diagnosed at term. Preeclampsia and CVD are conditions that share many risk factors, such as chronic hypertension, diabetes, and obesity. It is unknown if the future risk for CVD is due to shared risk factors affecting systemic inflammation and endothelial dysfunction or if preeclampsia by itself causes long-term effects on the cardiovascular system. Taken together, the many similarities between CVD and preeclampsia imply common pathological pathways, and therefore an interest for cardiovascular biomarkers in preeclampsia has emerged.

Angiotensin-converting enzyme 2 (ACE2) is a surface protein found on many cell types and is highly expressed in vascular endothelial cells. ACE2 is a part of the depressor arm in the renin-angiotensin system (RAS). It acts through the conversion of Angiotensin II (Ang II) into Ang (1–7), thereby hampering fibrosis, mediating vasodilation, lowering blood pressure, and repressing oxidative stress. Recently, ACE2 has also been a focus of attention since it acts as the main entry port to host cells for the severe acute respiratory syndrome coronavirus 2 (SARS-CoV-2). In addition to the cell surface-bound ACE2, the protein also exists in a soluble form in plasma after being cleaved from its membrane-bound domain. The functional role of soluble ACE2 is unclear, but increased levels and activity have been reported in CVD^3^. Furthermore, high plasma levels of ACE2 are also associated with an increased risk for major cardiovascular events in the general population. With these factors in mind, ACE2 has been proposed as a novel CVD biomarker^4^.

In summary, preeclampsia and CVD share multiple features and risk factors. ACE2 is of great interest concerning CVD, but information relating to ACE2 and preeclampsia development is scarce. This study aimed to investigate if circulating ACE2 are affected in mid-pregnancy for women later developing preeclampsia.

## Methods

We used the population-based Uppsala Biobank of Pregnant Women cohort, consisting of plasma samples from mid-pregnancy. Since 2009 the biobank has recruited pregnant women at their routine second trimester ultrasound at the Uppsala University Hospital, Sweden. Eligibility criteria are 18 years of age or above, with Swedish as a spoken language. If the woman chooses to participate, a venous blood sample and brief maternal demographic data are collected. For this nested case–control study, women with singleton pregnancies without chronic hypertension or other pre-existing diseases were eligible. At the time of blood sampling all women selected for this study had a healthy pregnancy. We identified all women in the biobank between 2009 and 2018, later developing preeclampsia according to the then existing Swedish definition of preeclampsia; de-novo hypertension (blood pressure ≥ 140/90 mmHg) and proteinuria (≥ 300 mg/24 h or protein/creatinine ratio ≥ 30 mg/mmol or ≥ 2+ on a dipstick test), after 20 gestational weeks (cases). If a woman had more than one pregnancy complicated by preeclampsia included in the biobank, we included the pregnancy with the earliest preeclampsia onset in this study. Women with no prior history of preeclampsia and who continued to have a healthy pregnancy (i.e., normotensive and no pregnancy complications) were included as controls. We matched the cases and controls one-to-one based on parity and body mass index (BMI). After reviewing the medical records to confirm a diagnosis of preeclampsia and excluding samples due to technical issues prior to the protein analysis, the final cohort consisted of *n* = 296 cases of preeclampsia and *n* = 333 controls. We further divided the study cohort into a preterm group, including those with preterm preeclampsia (diagnosis before 37 gestational weeks, *n* = 97) and a matched reference group (healthy pregnancies, *n* = 92), and a term group including term preeclampsia (diagnosis at or after 37 gestational weeks, *n* = 199) and a matched reference group (healthy pregnancies, *n* = 193). The Regional Ethical Review Board in Uppsala, Sweden, approved the collection of blood samples into the biobank (Dnr: 2007/181) and the nested case–control study (Dnr: 2018/251). All research was performed in accordance with the Declaration of Helsinki and all participants gave written informed consent.

We measured relative levels of circulating ACE2 with an immunoassay based on proximity extension assay technology (Olink PEA CVD-II panel; Uppsala, Sweden)^5^. The PEA is a multiplex assay for high throughput detection of proteins. First, a matched pair of antibodies linked to unique oligonucleotides bind to the target protein present in the sample, enabling the oligonucleotides to hybridize. Then, by adding DNA polymerase, a DNA amplicon is created from the oligonucleotides. The DNA Amplicon is thereafter quantified with real-time polymerase chain reaction. The generated data were expressed as relative quantification on the log2 scale of normalized protein expression (NPX) values. A high NPX value corresponds to a high protein concentration.

The Mann–Whitney *U* test was used to compare differences of NPX levels between groups. In addition, logistic regression models were applied to estimate the association between mid-pregnancy ACE2 levels and development of preeclampsia, with healthy pregnancies as the reference group. The models were adjusted for maternal age, parity (primiparous or multiparous), body mass index (BMI), systolic/diastolic blood pressure, and smoking in early pregnancy, as well as gestational week at sampling. Data are presented as medians with interquartile range, frequency (percent), or odds ratio (OR) with 95% confidence interval (CI). The statistical analyses were done with the software IBM SPSS Statistics version 27.

## Results

Clinical characteristics of the study population are presented in Table 1. The groups did not differ in maternal age or smoking habits. However, women who developed preeclampsia had higher blood pressure at their first antenatal visit compared with controls (*p* ≤ 0.001). In both groups, plasma samples were collected at 18 gestational weeks (median). In women with preeclampsia, the time of diagnosis was on average at 38 weeks and two days (median). Most women were diagnosed at term but 32.8% were diagnosed before 37 gestational weeks (preterm preeclampsia).

**Table 1:** Clinical characteristics of women in the study by development of preeclampsia.

	Preeclampsia (*n* = 296)	Controls (*n* = 333)
Maternal age (years)	29 (26–34)	30 (27–33)
Primiparous, *n* (%)	193 (65.2)	199 (59.9)
**Early pregnancy**		
Body mass index (kg/m^2^)	25.1 (22.6–29.4)	25.1 (22.6–29.1)
Smoking, *n* (%)	7 (2.4)	12 (3.6)
Systolic blood pressure (mmHg)*^§^	120 (113–128)	116 (110–123)
Diastolic blood pressure (mmHg)^†‡^	73 (66–80)	70 (65–77)
Gestational age at sampling (weeks)	18.4 (17.7–19.1)	18.3 (17.7–18.9)
Gestational age at delivery (weeks)*^||^	39.0 (37.6.-40.1)	40.2 (39.3–40.6)
Birth weight (g)*^||^	3270 (2830–3760)	3670 (3358–3990)
**Preeclampsia characteristics at diagnosis**		
Gestational weeks	38.3 (36.6–39.7)	
Preterm (< 37 weeks), *n* (%)	97 (32.8)	
Systolic blood pressure (mmHg)	150 (140–160)	
Diastolic blood pressure (mmHg)	95 (90–100)	

Data are given as medians (interquartile range) for continuous variables and frequency (%) for non-continuous variables. Differences between groups were tested with the Mann–Whitney *U* test or the Pearson’s chi-squared test, *p ≤ 0.001, ^†^*p* ≤ 0.01. Data are missing for three (^‡^), two (^§^), or one (^||^) participants.

**Figure 1:**
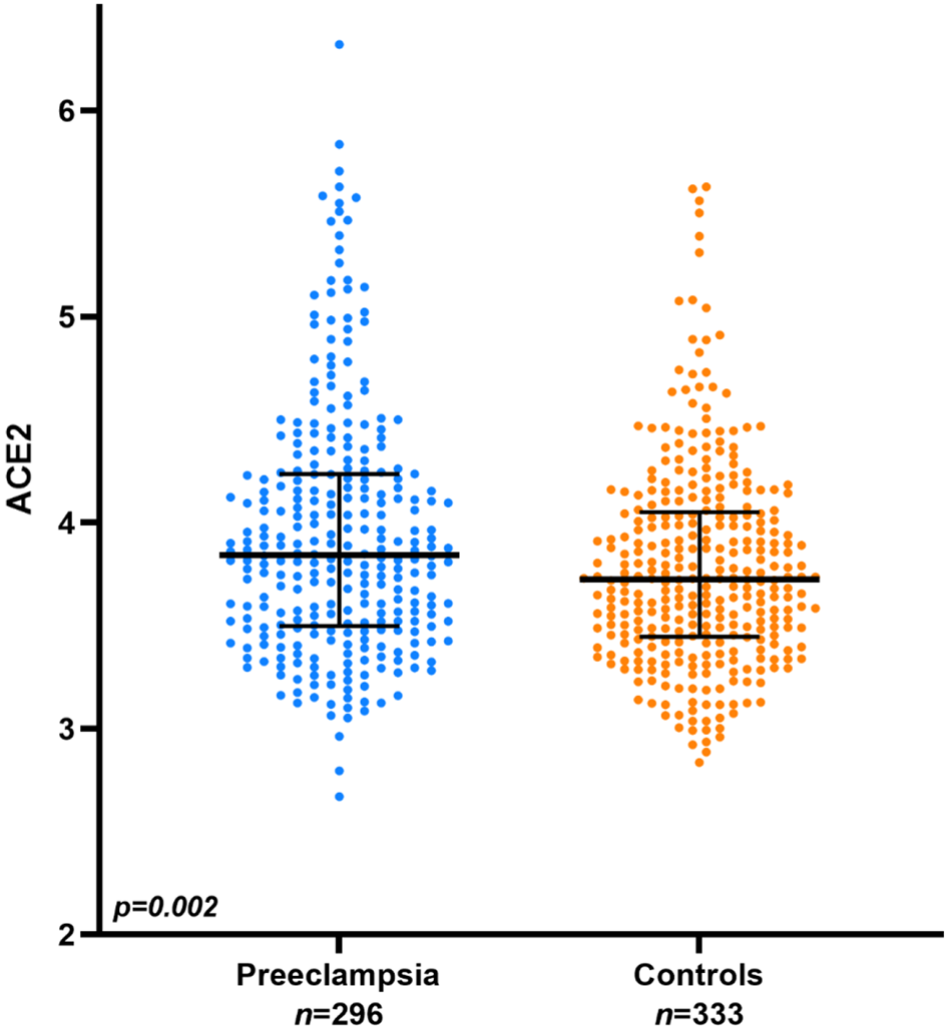
Relative circulating levels of angiotensin-converting enzyme 2 (ACE2) in mid-pregnancy. Horizontal lines represent medians and the interquartile range of ACE2 levels, *p* = 0.002 (Mann–Whitney *U* test).

Relative levels of circulating ACE2 were higher in women later developing preeclampsia than in controls; 3.84 (3.50–4.24) vs. 3.72 (3.45–4.04), *p* = 0.002 (Fig. 1). One unit elevation, corresponding to duplication of protein levels, of ACE2 was associated with a 70% increase in the odds for later development of preeclampsia (OR 1.70, 95% CI 1.27–2.28). When adjusting for maternal age, parity, BMI, smoking, and systolic/diastolic blood pressure at early pregnancy, and gestational weeks at sampling, the association was only slightly attenuated (OR 1.60, 95% CI 1.17–2.18). Preterm preeclampsia showed a stronger association with ACE2 than term preeclampsia. However, the 95% CI was wide and overlapping. In the preterm preeclampsia cohort, one unit elevation of ACE2 was associated with a 128% increase in the odds of preeclampsia (OR 2.28, 95% CI 1.27–4.10; adjusted OR 2.14, 95% CI 1.15–3.96). The corresponding increase in the odds of preeclampsia with elevated ACE2 in the term preeclampsia cohort was 48% (OR 1.48, 95% CI 1.03–2.11; adjusted OR 1.52, 95% CI 1.04–2.23).

## Discussion

We found increased ACE2 levels in women later developing preeclampsia compared to those who continued to have healthy pregnancies. To our knowledge, this is the first study of plasma ACE2 in mid-pregnancy before preeclampsia onset. There is only one previous report on plasma ACE2 and preeclampsia; in that study, women were examined after 28 gestational weeks, and the majority were included after the onset of preeclampsia. Contrary to the findings in our study, they report lower levels and activity of ACE2 in preeclampsia^6^. Another study found that plasma levels of Ang (1–7) were increased in normal pregnancy but reduced in women with preeclampsia at term^7^. This reduction could possibly be a consequence of lower activity of ACE2 since this enzyme converts Ang II into Ang (1–7). Our study adds new knowledge to the field and suggests that ACE2 is increased in mid-pregnancy in women developing preeclampsia. It could be that circulating levels of ACE2 are initially increased but later decrease in pregnancies complicated by preeclampsia. The cause behind this would need to be further explored. To test this hypothesis, it would be valuable to conduct a prospective longitudinal study of ACE2 in pregnant women, studying the dynamics of ACE2 during both healthy pregnancies and those complicated by preeclampsia.

Women who experienced preeclampsia have an increased risk of developing CVD later in life. This risk is even higher in women with preterm preeclampsia. Interestingly, we observed the strongest association between increased ACE2 levels and later development of preterm preeclampsia. Even if many of the functions of ACE2 are still unknown, the RAS-system is involved in CVD. The role of RAS in preeclampsia is presently a question for debate^8,9^. Our results suggest alterations in the depressor arm of RAS early in pregnancy for women who later develop preeclampsia. This indicates dysregulation of the RAS system, thereby possibly playing a part in the pathophysiology of preeclampsia. In fact, it has been proposed that increased ACE2 shedding and increased levels of circulating ACE2 could be a physiological stress response and that this system may be dysregulated in other pathological conditions such as CVD^10^. Since it is hypothesized that the cardiovascular system is dysregulated even before pregnancy in women developing preeclampsia, it would be interesting to study ACE2 levels prior to pregnancy and the risk of preeclampsia.

Apart from CVD, preeclampsia is also associated with COVID-19. In addition, a higher incidence of preeclampsia has been reported among those infected with SARS-CoV-2^11,12^.

The virus binds to membrane-bound ACE2 with high affinity, leading to a loss of its protective functions while shifting towards the classical arm of RAS. This, in turn, leads to fibrosis, oxidative stress, and vasoconstriction^13^. Interestingly, such features are shared by preeclampsia and CVD. It is hypothesized that increased plasma levels of ACE2 render a person more susceptible to SARS-CoV-2 infection^14^. In fact, it has been shown in vitro that the virus utilizes the soluble form of ACE2 to gain entry into host cells^15^. In the context of the ongoing COVID-19 pandemic, our novel finding of increased ACE2 levels in women who later develop preeclampsia is of interest. Our results suggest a previously unknown link between preeclampsia and SARS-CoV-2. If high ACE2 is a shared risk factor for preeclampsia and SARS-CoV-2 infection, this could partially explain the higher incidence of preeclampsia among women infected by SARS-CoV-2^11^. In fact, dysregulation of ACE2 was recently suggested to be involved in the development of preeclampsia, fetal growth restriction, and COVID-19-associated pregnancy pathologies^16^. Additional research on this common link could further expand understanding of preeclampsia, CVD, and COVID-19.

Our study has several strengths. The cohort is population-based, large, and clinically well characterized. We matched study groups in terms of BMI and parity, adjusting for covariates as potential confounders. There are also study limitations. First, the PEA multiplex method only allows for the measurement of relative and non-absolute protein levels. However, other studies have shown a good correlation between protein levels measured with the Olink PEA multiplex method and concentrations measured with well-established singleplex methods^17^. Secondly, increased membrane-bound ACE2 has protective effects, but it is still unclear if circulating ACE2 has the same properties, with the correlation between cell-bound and circulating levels being unknown. Moreover, we cannot conclude if the increased plasma ACE2 levels observed in our study are due to increased synthesis, decreased enzymatic cleavage, or decreased plasma clearance of ACE2. Third, catalytic activity instead of plasma levels of circulating ACE2 are often reported. A strong correlation between catalytic activity and the concentration of ACE2 has been reported in women with chronic hypertension^3^. It is unknown if this correlation is the same for pregnant women. The differences in ACE2 between the groups are interesting from a pathophysiological standpoint. However, since the measured levels are relative and somewhat overlapping between women developing preeclampsia and controls, the potential for clinical use is uncertain.

In conclusion, our finding of increased circulating ACE2 at mid-pregnancy points to dysregulation of the RAS system in women later developing preeclampsia, particularly preterm preeclampsia. Moreover, the results indicate a partly shared pathophysiological pathway between preeclampsia, CVD, and COVID-19 that need further exploration.

## Data Availability

The data that support the findings of this study are available from the corresponding author upon reasonable request.

## References

[1] Ghulmiyyah L, Sibai B (2012). Maternal mortality from preeclampsia/eclampsia. Semin. Perinatol..

[2] Leon LJ (2019). Preeclampsia and cardiovascular disease in a large UK pregnancy cohort of linked electronic health records: A CALIBER study. Circulation.

[3] Zhang Q (2018). Association of angiotensin-converting enzyme 2 gene polymorphism and enzymatic activity with essential hypertension in different gender: A case–control study. Medicine (Baltimore).

[4] Narula S (2020). Plasma ACE2 and risk of death or cardiometabolic diseases: A case–cohort analysis. Lancet.

[5] Assarsson E (2014). Homogenous 96-plex PEA immunoassay exhibiting high sensitivity, specificity, and excellent scalability. PLoS ONE.

[6] Tamanna S (2020). Angiotensin converting enzyme 2 (ACE2) in pregnancy: Preeclampsia and small for gestational age. Front. Physiol..

[7] Merrill DC, Karoly M, Chen K, Ferrario CM, Brosnihan KB (2002). Angiotensin-(1–7) in normal and preeclamptic pregnancy. Endocrine.

[8] Gathiram P, Moodley J (2020). The role of the renin-angiotensin-aldosterone system in preeclampsia: A review. Curr. Hypertens. Rep..

[9] Lumbers ER, Delforce SJ, Arthurs AL, Pringle KG (2019). Causes and consequences of the dysregulated maternal renin-angiotensin system in preeclampsia. Front. Endocrinol. (Lausanne)..

[10] Kuriakose J, Montezano AC, Touyz RM (2021). ACE2/Ang-(1–7)/Mas1 axis and the vascular system: Vasoprotection to COVID-19-associated vascular disease. Clin. Sci. (London, England)..

[11] Wei SQ, Bilodeau-Bertrand M, Liu S, Auger N (2021). The impact of COVID-19 on pregnancy outcomes: A systematic review and meta-analysis. CMAJ.

[12] Papageorghiou AT (2021). Preeclampsia and COVID-19: Results from the INTERCOVID prospective longitudinal study. Am. J. Obstet. Gynecol..

[13] Gheblawi M (2020). Angiotensin-converting enzyme 2: SARS-CoV-2 receptor and regulator of the renin-angiotensin system: Celebrating the 20th Anniversary of the Discovery of ACE2. Circ. Res..

[14] Sama IE (2020). Circulating plasma concentrations of angiotensin-converting enzyme 2 in men and women with heart failure and effects of renin-angiotensin-aldosterone inhibitors. Eur. Heart J..

[15] Yeung ML (2021). Soluble ACE2-mediated cell entry of SARS-CoV-2 via interaction with proteins related to the renin-angiotensin system. Cell..

[16] Tamanna S (2021). ACE2: A key modulator of the renin-angiotensin system and pregnancy. Am. J. Physiol. Regul. Integr. Comp. Physiol..

[17] Lekva T (2020). Multiplex analysis of circulating maternal cardiovascular biomarkers comparing preeclampsia subtypes. Hypertension.

